# Cerebral Organoid Research Ethics and Pinning the Tail on the Donkey

**DOI:** 10.1017/S0963180123000221

**Published:** 2023-10

**Authors:** Alex McKeown

**Affiliations:** Department of Psychiatry, Wellcome Centre for Ethics and Humanities, Warneford Hospital, Oxford, UK

**Keywords:** organoid research, consciousness, therapeutic benefits, suffering, research ethics

## Abstract

The risk of creating cerebral organoids/assembloids conscious enough to suffer is a recurrent concern in organoid research ethics. On one hand, we should, apparently, avoid discovering how to distinguish between organoids that it would be permissible (non-conscious) and impermissible (conscious) to use in research, since if successful we would create organoids that suffer. On the other, if we do not, the risk persists that research might inadvertently continue to cause organoids to suffer. Moreover, since modeling some brain disorders may require inducing stress in organoids, it is unclear how to eliminate the risk, if we want to develop effective therapies. We are committed to harm avoidance but hamstrung by a presumption that we should avoid research that might tell us clearly when suffering occurs. How can we negotiate this challenge and maximize the therapeutic benefits of cerebral organoid research? The author interrogates the challenge, suggesting a tentative way forward.

## Introduction

On balance and all-things-considered, we should use cerebral organoids and assembloids for research despite the risk that, in the case of assembloids in particular, we are likely to eventually create beings with some level of phenomenal consciousness and capacity for suffering. While non-ideal and anthropocentric, I defend the view that this is an acceptable price to pay, since sufficiently complex assembloids are the most likely to give us useful models for developing therapies that can successfully combat disease and illness in humans. For reasons on which I expand, since we do not know (yet) exactly under what conditions consciousness becomes present, until we do we are *guessing*; as such, the risk that we might inadvertently cause cerebral organoids to suffer in medical research must be accepted, however regrettably.

An ethical concern raised regarding the use of cerebral organoids relates to the risk that research might cause the organoids to suffer, because it cannot be ruled that they are, or may in future be, sufficiently conscious for such suffering to be possible.^1^ ^,^^2^ If this concern is reasonable, one potentially valuable avenue of cerebral organoid research appears to be, if not morally impermissible, then at least an ethical constraint on the terms of acceptability in such research; namely, deliberate investigations into the conditions under which consciousness—whether “merely” phenomenal or involving higher order capacities such as intentionality, metacognition, reflexivity, and so on^3^—arises. This is because if we succeed in engineering consciousness to discover the conditions required for its occurrence, the risk increases significantly that our experiments might (or might not, but we cannot yet know) cause suffering to the now conscious organoid, which is an outcome that we should ideally seek to avoid.^4^ ^,^^5^ If this is so, we should only carry out research that we have “sufficient” confidence will remain “beneath” the “threshold” of suffering, at which a nominal right to protection^6^ is activated; namely, when phenomenal consciousness occurs.

This poses a dilemma. On one hand, we cannot assume that it is ethically permissible to seek the information needed to distinguish between cerebral organoids that—we take it for now, at least—it would be permissible (i.e., non-conscious) and impermissible (i.e., conscious) to use for medical research. This is because, as Tsutomu Sawai and colleagues point out,^7^ presumably there are some categories of conscious experience, which cause such significant suffering to the organisms that studies involving them should be discontinued, where such suffering is shown to occur. On the other hand, if we do not do this, the risk persists that such organoid research might be accidentally harming them, since we do not know what the conditions are for their being sufficiently conscious that such suffering is taking place, nor how to measure it. We are committed to harm avoidance but should avoid pursuing that research, which might deliberately give us a clearer idea of when harm would and would not occur.

We are, in this respect, engaged in something resembling the game “Pin the tail on the donkey.” Here, the researcher is the blindfolded person; the donkey is the organoid or assembloid; the tail represents our research interventions, which carry a risk of harm if the organoid or assembloid is sufficiently conscious to suffer from them; successfully pinning the tail in the right place on the donkey is the suffering caused if the organoid or assembloid is sufficiently conscious to experience it. In the same way that the game in question is characterized by *guessing*, in doing research using organoids and assembloids we are guessing about whether we are causing them suffering, and we will continue to guess unless we know what conditions phenomenal consciousness requires.

Given the apparent unavoidability, one way or the other, of risking creating organoids and assembloids, which we then cause to suffer, how should we negotiate this obstacle to maximize the potential therapeutic benefits of brain organoid research? Here, I provide a tentative way forward. In doing so, I explore interconnected ethical and epistemic factors, drawing on analogies with ethics of animal research,^8^ ^,^^9^ artificial intelligence,^10^ ^,^^11^ and what panpsychist theory^12^ ^,^^13^ and part/whole relations^14^ ^,^^15^ might be able to tell us in responding to the challenge.

To begin, I consider ethical and epistemological implications of two possible courses of action: deliberately engineering consciousness so that suffering to organoids or assembloids with phenomenal consciousness can be avoided in the future (option A); and continuing with medical research using such organisms, despite the risk of creating those that might suffer in the course of that research (option B), and I defend a way forward taking these implications into account.

## Options A and B

Here are two options, given the objection to cerebral organoid research that it should not be pursued where it causes conscious organoids to suffer, assuming we could also measure this and agree about the measurements.

### Option A

Under this option, research would be done deliberately to try to establish the conditions under which phenomenal consciousness occurs. This is to say, we *attempt explicitly* to create such consciousness. However, this is done so that, having established what those conditions are, we can create subsequent cerebral organoids in the future, which remain beneath this threshold (here, again, for the purposes of argument, I make two assumptions that would require further clarification: namely, that “beneath” and “threshold” are accurate terms for describing how consciousness becomes present). So, having done the research necessary for identifying the conditions under which it is present, we could avoid causing suffering to future cerebral organoids. This would force us to do the thing that, it is claimed, we ought to avoid: creating cerebral organoids with phenomenal consciousness that are used in research. However, it would limit the number of conscious organoids that might suffer in research overall, since we would know how to avoid it in the future.

Nevertheless, despite this trade-off, option A is untenable for other reasons. First, it is, I argue, inevitable that organoid research, which is effective for treating certain kinds of brain-based disorder, will require the creation of assembloids, in particular, and may not completely eliminate the risk of creating organoid systems with the capacity for suffering. Second, in some instances, it may be necessary to create organoids with the capacity for suffering, where the development of a therapy for a given condition depends on inducing stress in the organism.

For these reasons, I contend that option B is a more realistic approach to this set of ethical challenges, all things considered.

### Option B

Under this option, we deliberately pursue research using cerebral organoids in the full knowledge that we *might* at some point create organoids or, as is more likely, assembloids,^16^ ^,^^17^ which achieve phenomenal consciousness, and we do not build into our research protocols the need to stop such research if this occurs. This is to say, we do not do the research to find out when consciousness is present, so that we can stop the research at that point. Rather, we take the risk that this is what will happen and continue the research, grounded in a justificatory basis on which this can be defended.

It is important to be clear that the approach I defend is not ethically straightforward and is open to criticism. It is vulnerable to the charge of speciesism, and the defense I make is a non-ideal, all-things-considered view, grounded in the primacy of developing therapies that can treat diseases in humans and alleviate human suffering. As such, I acknowledge the seriousness and legitimacy of the charges that can be made against my argument.

## Ethical Challenges

Here I address several salient ethical issues associated with the situation at hand.

### Biting the Bullet

Perhaps, all things considered, in trying to discern what we should do with respect to the possibility of phenomenal consciousness in cerebral organoids, we should just “bite the bullet” and accept that we cannot avoid the risk completely. By extension, therefore, we should also bite the bullet with respect to the possibility of causing suffering to such organisms.

Unfortunately, no research is completely risk-free, and all research involves trading off those risks with the attendant benefits.^18^ As such, it is unrealistic to believe that cerebral organoid research, done for the purpose of developing effective therapies for humans, could *completely* eliminate the risk of creating an organoid with phenomenal consciousness and the capacity to suffer. The seriousness of this should not be underestimated. To be clear, the position I advance is that the risk of causing suffering to these organisms is outweighed by the potential good that could be done by developing effective medical therapies for human disease and illness. This is to say, I do not regard the risk of causing suffering to organoids as morally unimportant. Rather, I acknowledge that the justification is grounded in a trade-off that is open to the objection that the risk of harm is *not* outweighed by the possible eventual therapeutic benefit.

### Organoid Research, Animal Research, and Speciesism

To push the justification a little further, an important part of it is grounded in a utility-driven perspective on research, with which we are already familiar. For instance, the scale of potential benefits to human health is adduced as one reason why, on balance, it is permissible to use certain animals, including some primates, in research.^19^ Here, the size of the benefit, in terms of the number of humans who stand to benefit, is deployed to legitimate—regrettably— harm and suffering caused to the animals,^20^ assuming the demand that certain conditions of treatment are met to minimize this. Indeed, as Bernard Rollin contends, however regrettable it is to use certain animals in medical research, and despite broad agreement that animal use is at least ethically problematic, there is no universal social consensus on how the suffering it causes should be completely avoided “short of abandoning the enterprise, which society is not in fact willing to do.”^21^

Of course, it does not follow from this that causing suffering to animals in medical research is ethically *un*problematic. The mere absence of consensus across society on what else to do does not neutralize the ethical objections. Certainly, any more effective alternative for developing therapies for human use would be preferable and should be sought,^22^ and this is acknowledged among those involved in such research.^23^ ^,^^24^ Moreover, there is disagreement about whether, overall, animal research is or is not the most reliable currently available approach to developing medical therapies for use in humans.^25^ ^,^^26^

Indeed, this non-ideal weighing of priorities and ethical commitments implicitly underlies the principles of the Three Rs.^27^ These stand for Replacement, Reduction, and Refinement, and were developed over 50 years ago in recognition of the suffering caused to animals used in medical research and provide a framework for making animal research as humane as possible. The principles support three commitments: aiming to replace animals with effective alternatives wherever possible, which includes developing novel techniques, one example of which is organoid research; minimizing the number of animals used in research; and minimizing the suffering caused by the refinement of experimental techniques.

Nevertheless, the claim that it is permissible in many circumstances to use animals that might suffer in the course of research into human drug development is open to the charge of speciesism^28^ ^,^^29^ in a way that is hard to completely refute. As such, it is important to state baldly that however the charge is couched, I am prepared to concede that I am putting human welfare ahead of animal welfare, in the absence of better alternatives in medical research.

With that in mind, I acknowledge the gravity of the charge that utility-driven arguments cannot justify the risk of causing cerebral organoids and assembloids to suffer. However, whether or not one objects to the argument is orthogonal to whether the analogy with *animal* research holds. If it does, why should we not regard cerebral organoids as consistent, with respect to what is, all-things-considered, ethically permissible? Perhaps one response is that the apparently particular moral significance in the cerebral organoid case follows from the risk that we might synthesize *human* phenomenal consciousness.^30^ After all, a key plank of the purported justification for experimentation on animals is precisely that they are *not human* ^31^ but are sufficiently biologically similar in certain respects for the experiments to potentially have value for the development of therapies for humans. If this line of reasoning holds, then creating conscious experience in *human* biological material would, presumably, transgress the key moral condition at stake in distinguishing which beings may and may not be experimented on.

This is a legitimate concern if we believe the rights we grant to humans are well-founded and coherent, relative to those we ascribe to animals which we claim we can justify overriding in the name of human health. There are grounds for skepticism, however. Just because a cerebral organoid is created from cloned *human* stem cells, it is unclear how similar any experience that it might have is of a fully developed human person with a body and the sophisticated kind of experience that is characteristic of them.^32^ Even an assembloid, where, for example, a cerebral organoid is integrated with a retinal organoid in an attempt to mimic the inputs, outputs, and feedback that comes from being “coupled” to the world with sense organs, would be significantly different from a human person. While systems of organoids might be created to usefully model sensorial inputs and proto-behavioral outputs, without a body, speech organs, a full suite of human-like experiences and behavioral options, cerebral organoids will still differ notably from human persons;^33^ this is to say, the kind of beings whose lives we are committed to improving through medical research.

What, then, can we conclude from all this? My suggestion is that, on balance, we should persist with using cerebral organoids in research *even if and in recognition of the possibility that* we might synthesize phenomenal consciousness, despite the risks associated with this.

### The Trajectory of Organoid and Assembloid Research

Implicit in the current limitations of cerebral organoid research is an incomplete understanding of the neural correlates of consciousness.^34^ ^,^^35^ The relation between brain and experience, while not in doubt, is not well understood with respect to what is required for the former to be capable of the latter.^36^ Reports on the state of the art in organoid research indicate that the direction of travel is increasingly sophisticated disease modeling, enabled by combining different tissue types implicated in the diseases and processes under study.

There is only so much that will be therapeutically achievable for brain-based disorders using discrete cerebral organoids in isolation.^37^ This is because it is characteristic of human persons to have experience of the external world via sense organs and limbs, which transmit information to the brain, which is essential for synthesizing experience and an awareness of the self as distinct from but existing in an objective external reality. I will discuss more in detail about the moral significance of experiential aspects of disease in the following section. However, for now it is sufficient to state that the accuracy of disease and brain modeling, and, by extension, the development of therapies will increase in line with how closely it is possible to replicate the systems which must be targeted by those therapies.^38^

Given the rationale directing this research trajectory, achieving greater accuracy in brain modeling for developing therapies will, as already mentioned, turn in part on the creation of *assembloids.* ^39^ ^,^^40^ ^,^^41^ ^,^^42^ This direction of travel, toward circuits of cerebral and other organoids, represents a logical direction for this research because an assembloid in which a cerebral organoid is coupled to the world via sensory inputs reflects basic facts about the causal components of phenomenal consciousness.^43^

For example, even if this possibility remains relatively distant at present, Bonsang Koo et al. pose the question of whether it is “possible to make a neural network in a brain organoid with computational ability, memory, and intelligence.”^44^ As a tentative answer to this question, they cite examples of research which has detected neural activity in organoids that is consistent with that observed in the preterm human brain; and demonstrates that photosensitive retinal neurons in a brain organoid could react to light stimuli.

Moreover, regarding the rationale for using cerebral assembloids, Alexios Panoutopoulos states that these circuits are “more sophisticated than in typical organoids,” creating an environment that is “more similar to the diverse environment of the brain” and as such has “greater resemblance to real conditions.”^45^

Consistent with this, Sergiu Paşca writes that “… there is a pressing need to develop functional, realistic and personalized models of the developing human brain so that we can better understand its unique biology and gain mechanistic insights into neuropsychiatric disorders.”^46^

In a later paper on the same subject, Paşca emphasizes that it is precisely *because* “understanding the mechanisms of psychiatric disorders will require more accurate models of the human brain” that “more discussions about the ethical implications will be needed.”^47^

Given this trajectory, there may be no way to guarantee that we can avoid breaking through the “threshold” of phenomenal consciousness and, therefore, the possibility of causing suffering. Again, to return to the assumed nomenclature of “threshold” mentioned earlier, I use the term advisedly here. Phenomenal consciousness may or may not be something that occurs at a threshold; it may be a capacity of degree, for example. As such, I use “threshold” only as a placeholder for the conditions, whatever they might be, under which suffering resulting from phenomenal consciousness is possible. Nevertheless, even if “threshold” is not the appropriate term, there is still a potential problem, given the reasons why we engage in cerebral organoid research at all, namely, the development of effective therapies for humans.

There is a logic to the way that this process of development unfolds. Somewhere between neuronal activation in a laboratory and a fully integrated human person, the experiences to which we ascribe value occur. As such, my conjecture is that since the sophistication required for this represents the direction of travel—necessarily, given that this is required for maximizing therapeutic effectiveness—we should accept the reality that we may find ourselves engineering phenomenal consciousness, *even if there is no deliberate intention to do so.*

Earlier, I mentioned the moral significance of experiential aspects of disease. These aspects are more likely to emerge in the context of assembloids, which can provide cerebral organoids with sensory inputs. In the next section, I outline why these experiential aspects are ethically pertinent to cerebral organoid research.

## Experiential Aspects of Disease

The feature of disease and illness that provides the primary normative impetus for research is the *experience* of disease and illness. If it were not for the harm and suffering caused by disease and illness, the ethical imperative for medical research would be absent. Of course, harm and suffering are not necessarily coterminous, since a disease can harm silently, without symptoms that cause suffering. In the brain, it is possible for there to be pathology that causes harm but not suffering, at least in the early stages of disease, for example, as in Alzheimer’s disease.^48^ Here, the moral case for research into pathology still holds, because incipient harm is occurring, but it does not yet share the other morally relevant feature caused by suffering once symptoms emerge.

Certainly, in pathology that *does* cause suffering, these experiential aspects cannot be subtracted from the picture with respect to what must be understood for therapies to treat those symptoms effectively. Again, we are already familiar with this in cases of stress research involving animals, where the *explicit aim* of the experimentation is to induce stress.^49^ ^,^^50^ These experiments can only be carried out on creatures that have a relatively sophisticated existence, with sense organs and corporeal features in virtue of which they can experience suffering in a way that could be translated into therapies for humans. Absent that coupling, observation of a stress response is limited or unreliable, since it does not replicate what would be happening in an animal in its environment when it receives the negative stimulus.

There are instances in the cerebral organoids context that underline why the experiential aspect cannot be subtracted from the picture, given that a key normative reason for engaging in medical research is to prevent or reduce suffering and the harm it does.

For example, cerebral organoids are being used in research investigating what occurs in the brain of people with post-traumatic stress disorder (PTSD).^51^ These experiments, while valuable, have limitations because, although they produce cortisol readouts to show what happens when stress is induced, there is no experiential aspect that can be *qualitatively* interpreted. This is important, because PTSD is a debilitating and serious condition that causes profound and persistent distress.^52^

Part of this distress follows from misinterpreting sensory inputs as dangerous, following earlier exposure to harm, even when danger no longer exists in the present.^53^ It involves numerous components, including the hippocampus and amygdala,^54^ the vagus nerve,^55^ and the modulation of neurochemicals.^56^ In people with PTSD, dysregulation of the systems involved can have a wide range of severe negative impacts on the capacity for normal living, including emotional difficulties, distortions of perception, impairment of memory, fear of relationships, intrusive thoughts, and sustained depression, among others.^57^ PTSD is a condition that explicitly involves *interpreting stimuli* from the external world, and effective treatment must therefore be able to mitigate misinterpretations and distortions of these stimuli.^58^ ^,^^59^ Therefore, without being able to model what happens in these systems and how they contribute phenomenologically to the experience of distress, the scope for developing effective therapies will be necessarily limited. Since consciousness cannot be measured in a non-behavioral entity, optimizing the effectiveness of organoid research for treating it will require creating assembloids, to reiterate an earlier point, which makes it more likely—although how likely is not yet known—that phenomenal consciousness will be achieved.

At this juncture, we can see that the ethical concerns intersect with uncertainty about what, if anything, is being experienced by organoids and how we could meaningfully interpret it. As such, there is not only a pertinent moral dimension but also an epistemological one.

## Epistemic Challenges

### The Ubiquity of Phenomenal Consciousness in Biological Organisms

It is almost certainly true that simple cerebral organoids do not have phenomenal consciousness. As such, the difference between disease modeling in humans and in cerebral organoids is vast. However, this is, in one sense, somewhat odd. Although it appears common sense to hold that a simple cerebral organoid is not conscious, viewed from another perspective there might be more reason to ask why it should *not* have phenomenal consciousness.

To illustrate this, we can adduce part of the arguments made for panpsychism (without actually having to commit to the thesis). In service of his arguments for panpsychism, Galen Strawson reverses the skepticism about the possibility of matter having the capacity for experience, by way of the claim that “the only thing we know absolutely for certain is that there is consciousness.”^60^ Likewise, David Chalmers holds that it should be counter-intuitive to doubt that matter might be experiencing: “Some say that consciousness is an ‘illusion,’ but I have little idea what this could even mean. It seems to me that we are surer of the existence of conscious experience than we are of anything else in the world.”^61^

As I have said, we do not need to commit to the panpsychist thesis to take these positions seriously. Strawson and Chalmers are right to highlight the ubiquity of conscious experience, even if we only restrict this to biological matter and resist the move to panpsychism proper. The notable prevalence of phenomenal consciousness in even relatively simple biological creatures^62^ suggests that we should not just dismiss out of hand the possibility of the same occurring in cerebral organoids, even if in some unimaginably primitive way that is inscrutable to us, as absurd or a non-problem. As such, I suggest this principle should be kept in mind as research involving cerebral organoids continues.

### Epistemic Dimensions of the Animal Research Analogy

We can return here to the earlier analogy with ethical dilemmas in animal research. There is a comparison to be drawn with animal rights debates, in which a to-and-fro of skepticism about capacity for suffering occurs. For example, when I go fishing, and a fish that I am trying to land resists my attempts to do so once it is on the hook, I have the distinct impression that it is *suffering*: this seems to be the only plausible interpretation of its unwillingness to be caught. Undoubtedly, there is a linguistic barrier here to conceptualizing the experience that the fish is having. It is not clear that “unwilling” is an accurate way to describe the preference that the fish displays, but it is one way of communicating my interpretation of its actions. However, it is undoubtedly anthropomorphic, since it is a word grounded in the experience of being human, not a fish.

As such, the debate about suffering persists and includes a view that is skeptical of interpretations such as mine, namely, that the observed suffering in certain animals maps onto our own experience.^63^ ^,^^64^ Here again, similar to the comparison with pinning the tail on the donkey, I am *guessing* that the fish is suffering, even though there is a respect in which I am qualitatively “blind” to that experience and cannot imagine what is over the horizon of the experience. If skepticism *is* warranted, therefore, interpreting the behavior as suffering may be the best guess we have about what is going on, but it is still possible that it does not resemble our own suffering. If so, perhaps our causing the reaction we observe is less morally questionable. In the interests of disclosure, I am dubious of these arguments and am inclined to think that the fish, for example, is indeed suffering. Moreover, despite—I admit—being unsure whether it is all things considered morally permissible, I still go fishing. Given this admission, I flag these arguments as legitimate premises for the basis of a defensible position against the moral permissibility of activities such as these.

Another morally relevant epistemological matter analogous to debates in animal rights concerns the sophistication of a being’s consciousness. For instance, part of what we take to be morally wrong about killing or maiming humans, in contrast to the non-human animals used in medical research, is that these actions threaten that person’s future, their awareness of their future, and whatever plans they might have for it.^65^ An awareness of oneself persisting over time, with values that one wishes to honor and goals one wishes to fulfill is an advanced type of conscious awareness that a cerebral organoid, even if it experiences anything at all, would be very unlikely to possess.

Typically, perhaps mistakenly, our attribution of moral status tends to track degrees of sentience, up to sapience.^66^ This is to say, by virtue of being sapient, humans often ascribe our own species with higher moral status than ants, for example, because of how our ability to anticipate the future, evaluate, judge, decide, and make plans for living generates the particular kind of sophisticated existence that has become characteristic of human existence. Tying moral status to cognitive sophistication like this is one strategy for arguing for a distinction between those creatures to which causing harm can—however regrettably—be justified, in view of the gradation of sophistication that occurs across animal species. Indeed, this kind of strategy is used to argue for permitting animals to suffer in medical research, if doing so were to further human health.^67^ Needless to say, however, it follows from this that it is more or less permissible to use some species than others: If cognitive sophistication confers moral status, it is certainly less permissible to use higher primates in research than it is to use fruit flies, and so on.

Given these complexities, in the organoid case, it is true that the model of justification I advance is imperfect; indeed, it is undoubtedly anthropically biased.^68^ Nevertheless, if we wish to advance human health and mitigate the harm done by disease and illness, we may need to do things that ideally we ought not to do and which, in an ideal world, we *would not* do. As such, despite gaps in our knowledge of “what it is like” to be a cerebral organoid, and even if we judge it too improbable that our actions could cause it to suffer, here again there is a “bullet-biting” scenario: If we avoid the risk of suffering completely, we will limit how much benefit to human health we can bring about.

The analogy discussed here was, of course, drawn with other biological beings. However, there is also an analogy with machine-based Artificial General Intelligence (AGI), if it is ever created, which may be relevant to the ethical conclusions that we can draw.

### An Analogy with AGI Research

In an AGI, the aim is to create an intelligent agent with something that, broadly speaking, resembles sapient-level human consciousness, but which is machine-based rather than biological. Crucially, here, a key epistemic barrier is how to interpret the experience of even a very cognitively sophisticated agent that is so differently physically instantiated.^69^ ^,^^70^ The barrier exists because it is unclear how we would properly interpret or comprehend the phenomenal consciousness of a machine-based agent that is disembodied and whose interactions with the world are alien to our own.

Even beneath sophisticated conscious awareness, when reflecting merely on one’s physical orientation within the external world, Tom Ziemke contends that this radical difference presents a major obstacle to our capacity to empathize, stating that “nothing is more alien to our life-form than a network with no up/down, front/back orientation, no interior/exterior distinction.”^71^ Likewise, when considering what it might be like to have representations of the world that are not informed by being able to view the environment or to move around within it, which might be the case for an AGI, Maja Mataric asks, “What might human nonspatial or nonmotor representations come from and look like?”^72^ The incomprehensibility to us of such a mode of experience underlines the difficulty we face in trying to determine both “where” consciousness might emerge and how to begin to grasp it in the case of other beings whose physical instantiation is radically different from our own.

Again, then, there is a choice to be made between resisting from engaging in research, which might inadvertently cause suffering and continuing to engage in the research, accepting both the risk of possibly causing suffering, and the difficulty of interpreting that suffering as such because of the radical difference in physical instantiation between an organoid and a fully integrated human.

This is the key ethical decision to be weighed with respect to the risk of research on organoids, which may have, or come to have, phenomenal consciousness. Before drawing my conclusions, I will make some remarks about how we should guide our decision-making, given both what we know and the gaps in our comprehension. In particular, these draw on the work by Peter Hacker and others on part/whole relations and the “mereological fallacy.”^73^ ^,^^74^

## Part/Whole Relations

If we wish to use cerebral organoids developed from human stem cells for solving problems in human health, neurochemical reduction will go only so far. To return to the PTSD case, this would be to say that stress hormone readings from a discrete organoid, while undoubtedly useful, will be limited in what they can tell us about the experience of disease and illness. This is because, for a cerebral organoid, the whole being is *just* the organoid. In humans, by contrast, the whole being is a complex organism coupled to the world in numerous ways, with a variety of organic sensory and motor inputs, which can interact with the brain in feedback loops, creating options for behavioral decision-making.

Here, we can apply work by Peter Hacker and others on part / whole relations, the “mereological fallacy,” and what they claim to be a pervasive conceptual error in the language of neuroscience and cognitive science.^75^ In short, the thesis is that whatever promise neuro-reductionism has for understanding brain diseases and psychological disorders, if our focus is too narrow, this will impoverish the potential scope of our understanding. Rather, the argument runs that what is epistemically and ethically pertinent must, and can only, be grounded in the whole being in which the disease occurs and in which it is experienced.^76^ For instance, to reiterate an earlier point, a cerebral organoid is a whole being, but without coupling it sensorily to the world, and extending its parameters, there is a limit to what we can learn from it about us.

Here, the mereological fallacy is relevant. According to Harry Smit and Peter Hacker,^77^ the fallacy is committed when one attributes something to a part of an entity that can be attributed only to the whole. For example, given the acute sense that one’s capacity for self-awareness and reflection is fundamentally seated within one’s head more than anywhere else—this is to say, where one’s *brain* is—it can *appear* to make a certain kind of sense that this is how one identifies oneself. However, the identity is *merely* apparent and straightforwardly false. If you or I *were our brains*, we would be around 15 cm tall, and we would weigh around 1.5 kg. Clearly, this is not the case, because, although we both *have* a brain, it is an organ of a whole body in which the full, integrated suite of human experience is instantiated. Only by virtue of the other organs and the body can the brain do what it can in terms of its key role in generating conscious experience of the kind that we have. As Smit and Hacker write:

…we cannot see unless the visual cortex is functioning normally, but we see with our eyes, not with our brain…we cannot walk unless the motor cortex of the brain is functioning normally, but that does not mean that we walk with our brain and that the brain is the organ for locomotion, as the legs are.^78^

Again, part of the challenge with respect to cerebral organoids and how we should treat them is that in these beings, discrete brain tissue is *all that they are.* The simpler and less integrated an organoid is with other organoids, the lower the chance of phenomenal consciousness and, by extension, the lower the chance of our causing suffering. But, equally, given that the moral significance of human diseases derives primarily from the suffering they cause to humans, an organoid’s simplicity is also what imposes limits on its usefulness. In light of this, I suggest that it will be prudent for guiding our choices over the ethical landscape of research involving cerebral organoids for us to retain a *holist* perspective. This will recognize that any experience resembling distinctively human experience will be sophisticated, complex, and necessarily characterized by a being with the apparatus for feedback with its environment. A holist approach has the benefit of consistency with human experience and keeps us focusing on what is required to effectively model, as far as possible, what happens for humans in the experience of disease and illness.

Of course, there are potential responses. There are, after all, other theories of and potential thresholds for consciousness, depending on how one understands it.^79^ For instance, Hacker’s approach, among others,^80^ may be described as a “global” theory of consciousness, as it describes and *requires* the whole being, coupled to the world. Given how little we know about consciousness, however, “local” theories, which set the bar for experience much lower,^81^ ^,^^82^ may be valid candidates, too. Nevertheless, whichever we find more plausible, until we understand more, we continue to pin the tail on the donkey with respect to whether we are acting on a cerebral organoid that has phenomenal consciousness.

Finally, we might still judge it impermissible to deliberately attempt to generate consciousness so that we can avoid doing research involving conscious organoids in the future. However, for reasons outlined, I argue that, on balance, even if we do not deliberately aim at it, we should accept the risk of it occurring as a natural byproduct of the research we are doing. This is because allowing for the risk of phenomenal consciousness is the price of being able to carry out research as close as possible to the threshold of sophistication required for developing optimally effective therapies for psychological disorders, brain disease, and illness in humans.

## Conclusion

My aim has been to plot a course that can balance competing commitments. To discern the right course of action in a profoundly non-ideal situation, it is necessary to be pragmatic without being fatalistic, keeping a focus on the moral imperative to develop effective therapies for humans without losing sight of why we may also have obligations to cerebral organoids, in particular, vis-à-vis the possibility that we may eventually create sufficiently sophisticated iterations with phenomenal consciousness and the capacity for suffering in the purposes to which we put them.

My conclusion is therefore problematic. I conclude that, all things considered, we cannot avoid biting the bullet with respect to the risk that we might cause cerebral organoids or assembloids to suffer. Undoubtedly, this conclusion is anthropocentric and suboptimal, but if we wish to do what we can to mitigate the harm caused by disease and illness in humans, there is no straightforward way off the horns of the dilemma.

For the reasons I have outlined, I contend that we are somewhat blindly “pinning the tail on the donkey,” mostly because of the inscrutability of subjective experience, not least in creatures so radically different and less sophisticated than we as cerebral organoids, whether in isolation or as assembloids. Given that inscrutability, I conclude that we will continue in that direction for some time. However, the trajectory of medical research using cerebral organoids aims toward eventually modeling something sufficiently close to human experience that it can contribute to the development of effective treatments for psychological, psychiatric, and neurological conditions in humans.

As such, even if we do not *deliberately* seek to engineer phenomenal consciousness, given sufficiently sophisticated assembloids, and in light of the surprising ubiquity of such a capacity in biological organisms, there is a significant possibility that we will engineer it, even if only as a byproduct of our research into treating disease and illness in humans.

## Notes

1.

de Jongh D, Massey EK , Berishvili E , Fonseca LM , Lebreton F , Bellofatto K , et al. Organoids: A systematic review of ethical issues. Stem Cell Research & Therapy 2022;13:1–21 35870991 10.1186/s13287-022-02950-9PMC9308907.

2.

Chapman AR . Brain models in a dish: Ethical issues in developing brain organoids. AJOB Neuroscience 2019;10(3):113–1531361201 10.1080/21507740.2019.1632963.

3.

Rosenthal DM . How many kinds of consciousness? Consciousness and Cognition 2002;11(4):653–512470629 10.1016/s1053-8100(02)00017-x.

4.

Lavazza A , Massimini M. Cerebral organoids: Ethical issues and consciousness assessment. Journal of Medical Ethics 2018;44(9):606–1029491041 10.1136/medethics-2017-104555.

5. Jeziorski J, Brandt R, Evans JH, Campana W, Kalichman M, Thompson E, et al. Brain organoids, consciousness, ethics and moral status. *Seminars in Cell & Developmental Biology* 2022;144:97–102 available at https://www.sciencedirect.com/science/article/pii/S1084952122000866 (last accessed 10 February 2023).

6.

Levy N , Savulescu J. Moral significance of phenomenal consciousness. Progress in Brain Research 2009;177:361–7019818914 10.1016/S0079-6123(09)17725-7.

7.

Sawai T , Sakaguchi H , Thomas E , Takahashi J , Fujita M. The ethics of cerebral organoid research: Being conscious of consciousness. Stem Cell Reports 2019;13(3):440–731509736 10.1016/j.stemcr.2019.08.003PMC6739740.

8.

DeGrazia D , Beauchamp TL . Beyond the 3 Rs to a more comprehensive framework of principles for animal research ethics. ILAR Journal 2019;60(3):308–1710.1093/ilar/ilz011PMC863344931598694.

9.

DeGrazia D. The ethics of animal research: What are the prospects for agreement? Cambridge Quarterly of Healthcare Ethics 1999;8(1):23–34 9924615 10.1017/s0963180199801054.

10. Hildt E. The prospects of artificial consciousness: Ethical dimensions and concerns. *AJOB Neuroscience*; 2022 Nov 21; available at https://www.doi.org/10.1080/21507740.2022.2148773 (last accessed 17 Mar 2023).

11.

Lyons S. Machine consciousness: Ethics and implications. In: Death and the Machine. Singapore: Palgrave Pivot; 2018:27–48 .

12. Chalmers DJ. *Panpsychism and Panprotopsychism. The Amherst Lecture in Philosophy*, Lecture 8; 2013; available at http://www.amherstlecture.org/chalmers2013/ (last accessed 17 Mar 2023).

13.

Strawson G. Real naturalism. Proceedings and Addresses of the American Philosophical Association 2012;86(2):125–54.

14.

Smit H , Hacker PMS . Seven misconceptions about the mereological fallacy: A compilation for the perplexed. Erkenntnis 2014;79(5):1077–97.

15.

Hacker PMS . Before the mereological fallacy: A rejoinder to Rom Harré. Philosophy 2013;88(1):141–8.

16.

Kanton S , Paşca SP . Human assembloids. Development 2022;149(20):dev20112010.1242/dev.20112036317797.

17.

Vogt N. Assembloids. Nature Methods 2021;18(1):27 10.1038/s41592-020-01026-x33408387.

18.

Miller FG , Joffe S. Limits to research risks. Journal of Medical Ethics 2009;35(7):445–919567696 10.1136/jme.2008.026062.

19.

Rollin BE . The ethics of animal research: Theory and practice. In: Kalof L , ed. The Oxford Handbook of Animal Studies. Oxford: Oxford University Press; 2017:345–63.

20.

Beauchamp TL , Frey RG , eds. The Oxford Handbook of Animal Ethics. Oxford: Oxford University Press; 2011 .

21.

Rollin BE . Reasonable partiality and animal ethics. Ethical Theory and Moral Practice 2005;8:105–21, at 10610.1007/s10677-005-3297-116459400.

22.

Kolar R. Animal experimentation. Science and Engineering Ethics 2006;12(1):111–2216501652 10.1007/s11948-006-0011-1.

23.

Baumans V. Use of animals in experimental research: An ethical dilemma? Gene Therapy 2004;11(1):S64–6 15454959 10.1038/sj.gt.3302371.

24.

Doke SK , Dhawale SC . Alternatives to animal testing: A review. Saudi Pharmaceutical Journal 2015;23(3):223–926106269 10.1016/j.jsps.2013.11.002PMC4475840.

25.

Garattini S , Grignaschi G. Animal testing is still the best way to find new treatments for patients. European Journal of Internal Medicine 2017;39:32–527916437 10.1016/j.ejim.2016.11.013.

26.

Akhtar A. The flaws and human harms of animal experimentation. Cambridge Quarterly of Healthcare Ethics 2015;24(4):407–1926364776 10.1017/S0963180115000079PMC4594046.

27. The 3Rs; 2017 available at https://nc3rs.org.uk/who-we-are/3rs (last accessed 17 Mar 2023).

28.

Horta O. What is speciesism? Journal of Agricultural and Environmental Ethics 2010;23:243–66.

29.

Steinbock B. Speciesism and the idea of equality. Philosophy 1978;53(204):247–56.

30.

Lavazza A. Human cerebral organoids and consciousness: A double-edged sword. Monash Bioethics Review 2020;38:105–2832895775 10.1007/s40592-020-00116-yPMC7723930.

31.

Frey RG . Justifying animal experimentation. *Society* 2002;39(6):37–47 .

32.

Andrews MG , Kriegstein AR . Challenges of organoid research. Annual Review of Neuroscience 2022;45:23–39 10.1146/annurev-neuro-111020-090812PMC1055994334985918.

33.

Fins JJ . Brain organoids and consciousness: Late night musings inspired by Lewis Thomas. Cambridge Quarterly of Healthcare Ethics 2021;30(4):557–6034702402 10.1017/S0963180121000037.

34.

Koch C , Massimini M , Boly M , Tononi G. Neural correlates of consciousness: Progress and problems. Nature Reviews Neuroscience 2016;17(5):307–2127094080 10.1038/nrn.2016.22.

35.

Aru J , Bachmann T , Singer W , Melloni L. Distilling the neural correlates of consciousness. Neuroscience and Biobehavioral Reviews 2012;36(2):737–4622192881 10.1016/j.neubiorev.2011.12.003.

36. Lepauvre A, Melloni L. The search for the neural correlate of consciousness: Progress and challenges. *Philosophy and the Mind Sciences* 2021;2; available at https://philosophymindscience.org/index.php/phimisci/article/view/9151 (last accessed 17 Mar 2023).

37.

Lancaster MA, Knoblich JA . Organogenesis in a dish: Modeling development and disease using organoid technologies. Science 2014;345(6194):124712510.1126/science.124712525035496.

38.

Farahany NA , Greely HT , Hyman S , Koch C , Grady C , Pasca SP , et al. The ethics of experimenting with human brain tissue. Nature 2018;556(7702):429–3229691509 10.1038/d41586-018-04813-xPMC6010307.

39.

Andersen J , Revah O , Miura Y , Thom N , Amin ND , Kelley KW , et al. Generation of functional human 3D cortico-motor assembloids. Cell 2020;183(7):1913–29.e2633333020 10.1016/j.cell.2020.11.017PMC8711252.

40. See note 16, Kanton, Paşca 2022.

41.

Pașca SP . The rise of three-dimensional human brain cultures. Nature 2018;553(7689):437–4529364288 10.1038/nature25032.

42.

Tambalo M , Lodato S. Brain organoids: Human 3D models to investigate neuronal circuits assembly, function and dysfunction. Brain Research 2020;1746:14702810.1016/j.brainres.2020.14702832717276.

43.

Panoutsopoulos AA . Organoids, assembloids, and novel biotechnology: Steps forward in developmental and disease-related neuroscience. The Neuroscientist 2021;27(5):463–7232981451 10.1177/1073858420960112.

44.

Koo B , Choi B , Park H , Yoon KJ . Past, present, and future of brain organoid technology. Molecules and Cells 2019;42(9):617 31564073 10.14348/molcells.2019.0162PMC6776157.

45. See note 43, Panoutopoulos 2021, at 468.

46. See note 41, Paşca 2018, at 437.

47.

Paşca SP . Assembling human brain organoids. Science 2019;363(6423):126–7, at 12730630918 10.1126/science.aau5729.

48.

McKeown A , Malhi GS , Singh I. Ethics of early intervention in Alzheimer’s disease. AJOB Neuroscience 2021;12(4):212–2333769219 10.1080/21507740.2021.1896595.

49.

Török B , Sipos E , Pivac N , Zelena D. Modelling posttraumatic stress disorders in animals. Progress in Neuro-Psychopharmacology and Biological Psychiatry 2019;90:117–3330468906 10.1016/j.pnpbp.2018.11.013.

50.

Heimbürge S , Kanitz E , Otten W. The use of hair cortisol for the assessment of stress in animals. General and Comparative Endocrinology 2019;270:10–730287191 10.1016/j.ygcen.2018.09.016.

51.

Hamilton KA , Santhakumar V. Current ex vivo and in vitro approaches to uncovering mechanisms of neurological dysfunction after traumatic brain injury. Current Opinion in Biomedical Engineering 2020;14:18–24 32548365 10.1016/j.cobme.2020.05.001PMC7297186.

52.

Yehuda R , Hoge CW , McFarlane AC , Vermetten E , Lanius RA , Nievergelt CM , et al. Post-traumatic stress disorder. Nature Reviews Disease Primers 2015;1(1):1–22 10.1038/nrdp.2015.5727189040.

53.

Kirkpatrick HA , Heller GM . Post-traumatic stress disorder: Theory and treatment update. The International Journal of Psychiatry in Medicine 2014;47(4):337–4625084856 10.2190/PM.47.4.h.

54. Akiki TJ, Averill CL, Wrocklage KM, Schweinsburg B, Scott JC, Martini B, et al. The association of PTSD symptom severity with localized hippocampus and amygdala abnormalities. *Chronic Stress* 2017;1. doi:10.1177/2470547017724069.

55.

Noble LJ , Gonzalez IJ , Meruva VB , Callahan KA , Belfort BD , Ramanathan KR , et al. Effects of vagus nerve stimulation on extinction of conditioned fear and post-traumatic stress disorder symptoms in rats. Translational Psychiatry 2017;7(8):e1217 28892066 10.1038/tp.2017.191PMC5611754.

56.

Sherin JE , Nemeroff CB . Post-traumatic stress disorder: The neurobiological impact of psychological trauma. Dialogues in Clinical Neuroscience 2011;13(3):263–7822034143 10.31887/DCNS.2011.13.2/jsherinPMC3182008.

57. Shalev A, Liberzon I, Marmar C. Post-traumatic stress disorder. *New England Journal of Medicine* 2017;376(25):2459–69.

58.

Yule W , Smith P. Post traumatic stress disorder. In: Thapar A , Pine DS , Leckman JF , Scott S , Snowling MJ , Taylor E , eds. Rutter’s Child and Adolescent Psychiatry. 6th ed. Oxford: Wiley; 2015:806–21.

59.

Lanius RA , Bluhm RL , Frewen PA . How understanding the neurobiology of complex post‐traumatic stress disorder can inform clinical practice: A social cognitive and affective neuroscience approach. Acta Psychiatrica Scandinavica 2011;124(5):331–4821854369 10.1111/j.1600-0447.2011.01755.x.

60. Strawson G. What is it like to be an electron? An interview with Galen Strawson by Robert Wright. *Nonzero* 2020 June 28; available at https://nonzero.org/post/electron-strawson (last accessed 17 Mar 2023).

61. See note 12, Chalmers 2013, at 2.

62.

Tononi G , Koch C. Consciousness: Here, there and everywhere? Philosophical Transactions of the Royal Society B: Biological Sciences 2015;370(1668):2014016710.1098/rstb.2014.0167PMC438750925823865.

63.

Yue Cottee S. Are fish the victims of ‘speciesism’? A discussion about fear, pain and animal consciousness. Fish Physiology and Biochemistry 2012;38:5–15 21086041 10.1007/s10695-010-9449-9.

64.

Jones RC . Speciesism and human supremacy in animal neuroscience. In: Johnson LSM , Fenton A , Shriver A , eds. Neuroethics and Nonhuman Animals. Cham, Switzerland: Springer; 2020:99–115 .

65.

Cohen C. The case for the use of animals in research. New England Journal of Medicine 1986;315(14):865–703748104 10.1056/NEJM198610023151405.

66.

Arnason G. The emergence and development of animal research ethics: A review with a focus on nonhuman primates. Science and Engineering Ethics 2020;26(4):2277–9332350758 10.1007/s11948-020-00219-zPMC7417401.

67.

Russow LM . Bioethics, animal research, and ethical theory. ILAR Journal 1999;40(1):15–21 11533512 10.1093/ilar.40.1.15.

68. See note 64, Jones 2020.

69.

McKeown A. What do we owe to novel synthetic beings and how can we be sure? Cambridge Quarterly of Healthcare Ethics 2021;30(3):479–9134109928 10.1017/S0963180120001036.

70. Bélisle-Pipon JC, Monteferrante E, Roy MC, Couture V. Artificial intelligence ethics has a black box problem. *AI & Society* 2022 Jan 5; available at https://doi.org/10.1007/s00146-021-01380-0 (last accessed 17 Mar 2023).

71.

Ziemke T. The construction of ‘reality’in the robot: Constructivist perspectives on situated artificial intelligence and adaptive robotics. Foundations of Science 2001;6(1):163–233, at 164.

72.

Matarić MJ . Studying the role of embodiment in cognition. Cybernetics and Systems 1997;28(6):457–70, at 460.

73. See note 15, Hacker 2013.

74.

Smit H , Hacker PMS . Seven misconceptions about the mereological fallacy: A compilation for the perplexed. Erkenntnis 2014;79(5):1077–97.

75.

Hacker PMS . The conceptual framework for the investigation of emotions. International Review of Psychiatry 2004;16(3):199–208 15511749 10.1080/09540260400003883.

76.

Bennett MR , Hacker PMS . On explaining and understanding cognitive behaviour. Australian Journal of Psychology 2015;67(4):241–50.

77. See note 74, Smit, Hacker 2014.

78. See note 74, Smit, Hacker 2014, at 1089.

79.

Seth AK , Bayne T. Theories of consciousness. Nature Reviews Neuroscience 2022;23(7):439–5235505255 10.1038/s41583-022-00587-4.

80.

Friston K. The free-energy principle: A unified brain theory? Nature Reviews Neuroscience 2010;11(2):127–3820068583 10.1038/nrn2787.

81.

Shevlin H. Non‐human consciousness and the specificity problem: A modest theoretical proposal. Mind and Language 2021;36(2):297–314 .

82.

Wiese W. The science of consciousness does not need another theory, it needs a minimal unifying model. Neuroscience of Consciousness 2020;2020(1):niaa01310.1093/nc/niaa013PMC735249132676200.

